# The Effect of a Rapid Heating Rate, Mechanical Vibration and Surfactant Chemistry on the Structure–Property Relationships of Epoxy/Clay Nanocomposites

**DOI:** 10.3390/ma6083624

**Published:** 2013-08-20

**Authors:** Betime Nuhiji, Darren Attard, Gordon Thorogood, Tracey Hanley, Kevin Magniez, Jenny Bungur, Bronwyn Fox

**Affiliations:** 1 Institute for Technology Research and Innovation (ITRI), Deakin University, Pigdons Road, Waurn Ponds, Geelong 3217, Australia; E-Mails: kevin.magniez@deakin.edu.au (K.M.); jenny.bungur@deakin.edu.au (J.B.); bronwyn.fox@deakin.edu.au (B.F.); 2 Australian Nuclear Science and Technology Organisation, PMB 1, Menai NSW 2234, Australia; E-Mails: darren.j.attard@gmail.com (D.A.); gjt@ansto.gov.au (G.T.); tha@ansto.gov.au (T.H.)

**Keywords:** nano-structures, vibration, mechanical properties, rheological properties, X-ray diffraction, small angle X-ray scattering, electron microscopy

## Abstract

The role of processing conditions and intercalant chemistry in montmorillonite clays on the dispersion, morphology and mechanical properties of two epoxy/clay nanocomposite systems was investigated in this paper. This work highlights the importance of employing complementary techniques (X-ray diffraction, small angle X-ray scattering, optical microscopy and transmission electron microscopy) to correlate nanomorphology to macroscale properties. Materials were prepared using an out of autoclave manufacturing process equipped to generate rapid heating rates and mechanical vibration. The results suggested that the quaternary ammonium surfactant on C30B clay reacted with the epoxy during cure, while the primary ammonium surfactant (I.30E) catalysed the polymerisation reaction. These effects led to important differences in nanocomposite clay morphologies. The use of mechanical vibration at 4 Hz prior to matrix gelation was found to facilitate clay dispersion and to reduce the area fraction of I.30E clay agglomerates in addition to increasing flexural strength by over 40%.

## 1. Introduction

Amongst the various classes of nano-materials that have been reported over the past few decades, polymer/clay nanocomposites have undoubtedly been the most studied and have shown the greatest potential for property improvements [1,2,3,4,5,6,7,8,9]. Even at low nanofiller weight percent, improvements in optical clarity [10], flame retardancy [11,12], thermo-mechanical [5] and gas and liquid barrier properties [13,14] have been demonstrated.

The degree of penetration of polymer molecules into the silicate clay galleries gives rise to different types of polymer/clay nanocomposites, classified according to both the distribution of layers and their homogeneity in the matrix. The term, exfoliated, refers to nanocomposites where the platelets of the clay are homogeneously suspended within the matrix. The term, intercalated, refers to nanocomposites where penetration of the polymer chains into clay galleries only causes an expansion of the platelets on the order of 1 to 4 nm. Additional descriptors, such as ordered and disordered, have been employed to further describe the micro-organisation of the platelets [9].

Of the research conducted on epoxy/clay nanocomposites, it has been determined that complete and homogeneous dispersion of the clay platelets within epoxy [2,9] is the key to achieving enhanced properties. The discrepancies in the literature indicate that the structure property relationships in these systems are not obvious [15]. For instance, tensile properties have been shown to increase when there is an exfoliated clay structure [1,16,17], although intercalated clay morphologies were reported to enhance toughness properties in other systems [18,19].

The main source of difficulty in achieving full exfoliation of nanoclays within epoxy resin systems arises from the technical difficulties in optimising processing conditions. Lou *et al.* successfully used an electromagnetic vibration technique (80 μm amplitude) to disperse CaCO_3_ nanoparticles in high density polyethylene during extrusion [20]. This process enhanced the shear flow field of the polymer, broke down agglomerate sizes and led to enhancements in both tensile and impact properties. The dispersion of clay structures into epoxies using ultrasonic vibration has also been reported [21,22,23], showing some promising results. It has also been reported that exposure to mechanical vibration during curing of epoxies improves the shear flow of the resin through a fibre preform by up to 60% [24], as well as reducing void content by employing low frequency pulses [25]. The ability to facilitate dispersion using this technique in conjunction with a low cost manufacturing method to control nanoclay morphology (technology-structure relationships) is of obvious interest for industry.

Previously, the present authors had studied the effects of mechanical mixing and alternating heating rates on the structure and properties of epoxy/montmorillonite (MMT) clay nanocomposites [6]. The combination of a high amplitude sonication method combined with a heating rate of 10 °C/min was shown to reduce the viscosity, which, in turn, facilitated the penetration of polymer chains in between clay platelets (I.30E organoclays). Clay *d*-spacings were measured at 142 Å when optimised processing conditions were used, although micron-sized agglomerates were observed in the nanocomposite.

In this work, by using the same high heating rate (10 °C/min) and sonication conditions that were previously reported [6], we extended the concept to investigate the effect that mechanical vibration (conducted during the heat up stage) and surfactant chemistry has on the dispersion of two commercial organoclays. Nanocomposites were characterised using X-ray diffraction (XRD), small angle X-ray scattering (SAXS), transmission electron microscopy (TEM) and optical microscopy methods, where the micro- and nanoscale morphologies of these materials were correlated to their respective mechanical and thermo-mechanical properties. The effect of surfactant chemistry on the epoxy-clay interface and the resulting differences in dispersion [in terms of clay *d*-spacing and the area fraction (%) of clay agglomerates] will also be discussed.

## 2. Results and Discussion

Two organoclays with various surfactant chemistries were investigated in this study. Table 1 outlines the differences in the chemical structures of the clay modifiers in I.30E and C30B clays. The difference in surfactant chemistries on the clays may lead to various morphologies in the epoxy nanocomposite, due to potential interfacial interactions between the surfactant and the epoxy during the curing process. This paper shows that sufficient evidence is required to characterise clay dispersion, where complementary diffraction, scattering and imaging techniques must be employed to characterise these morphologies. All of these characterisation methods have benefits and limitations, although they are complementary when combined and interpreted correctly.

**Table 1:** Organoclays and their alkylammonium surfactants.

Montmorillonite Organoclay	Organic Surfactant	
	Type	Structure
Nanomer I.30E	Octadecylamine	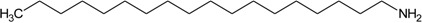
Cloisite C30B	Quaternary methyl, tallow, bis-2-hydroxyethyl, ammonium	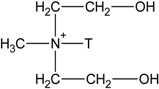

XRD and SAXS techniques are utilised to measure the clay gallery distances (*d*-spacing) between platelets by locating the position of the (001) and (002) crystallisation peaks. The shape and intensities of these peaks are used to examine the clay morphology in the nanocomposite. As XRD can measure interplanar spacings below 44 Å (located at 2° (2*θ*)) [26], SAXS is a corresponding tool that detects basal reflections at angles below 0.1 (Å^−1^) and, therefore, identifying whether clay gallery distances are greater than 44 Å. X-ray techniques are averaging methods, and therefore, bulk materials containing inhomogeneous clay morphologies are difficult to characterise by solely employing these methods.

Imaging techniques, such as TEM and optical microscopy combined with the X-ray methods, are important, as they provide photographic evidence at localised and spatial regions of the nanocomposites. TEM captures micrographs of individual clay platelets, whereas clay agglomerates observed at the micron scale are located on an optical microscope.

### 2.1. X-ray Diffraction, Small Angle X-ray Scattering and Transmission Electron Microscopy

**Figure 1:**
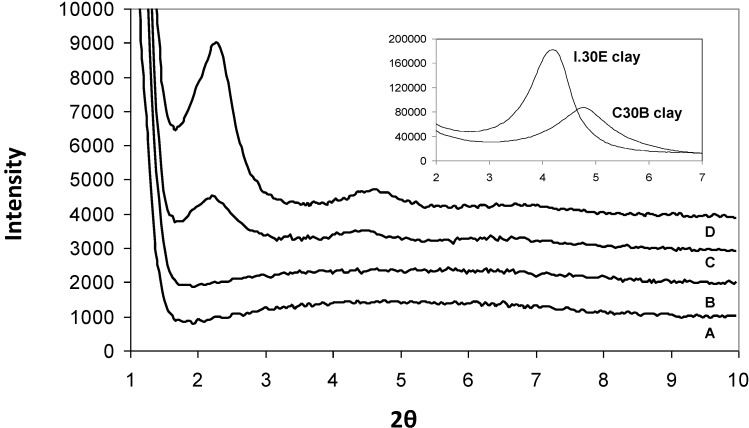
An X-ray diffraction (XRD) diffractogram of the epoxy/clay nanocomposites (**A**) I.30E_10MV; (**B**) I.30E_10; (**C**) C30B_10MV; and (**D**) C30B_10.

The XRD spectrum for the I.30E and C30B clays in their original powder form are shown in the top right corner of Figure 1. The basal *d*-spacing of the I.30E clay (21 Å) was found to be slightly higher than that of the C30B clay (19 Å), which is consistent with the literature [27]. The peak height measured for the I.30E clay is greater in intensity, suggesting an ordered clay orientation.

XRD results for the epoxy/clay nanocomposites were collated and shown in Figure 1. When the resin is combined with C30B clay and cured at a heating rate of 10 °C/min (C30B_10, Figure 1D), the nanocomposite clay structure exhibits a (001) basal peak reflection at 2.35° (2*θ*). The (001) diffraction peak shifts slightly to a lower angle of 2.24° (2*θ*) with the exposure of a 4 Hz mechanical vibration during curing (C30B_10MV, Figure 1C). The *d*-spacing values are similar (38 Å and 39 Å) and relate to the presence of what is commonly classified as an intercalated clay structure. The (002) basal reflections were also registered for both nanocomposites at ≈4° (2*θ*), although the intensity in the reflection peaks differs. The C30B_10MV nanocomposite exhibits a decrease in intensity, indicating that the exposure to mechanical vibration during curing may shear the C30B clay platelets in the resin to form a more disordered clay morphology. Conversely, I.30E nanocomposites did not register ((001) or (002)) diffraction peaks, implying that the clay morphologies in these nanocomposites are exfoliated.

**Figure 2:**
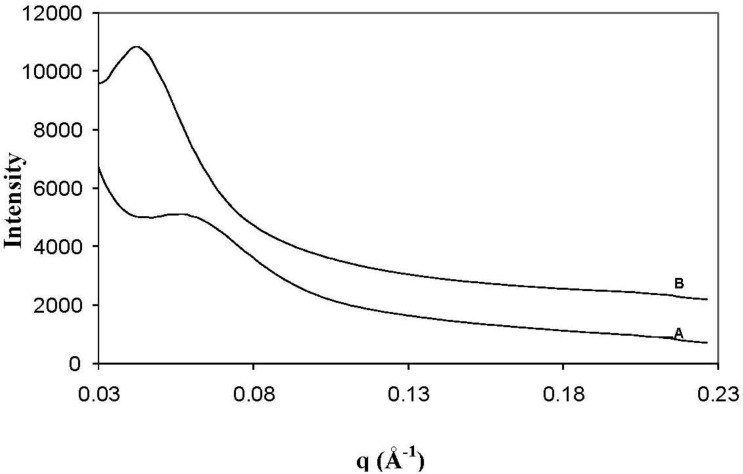
Small angle X-ray scattering data of the I.30E_10MV (**A**); and the I.30E_10 (**B**) nanocomposites.

SAXS was utilised to measure the *d*-spacing of the clay in the I.30E nanocomposites, as presented in Figure 2. From this figure, *d*-spacing values of 108 Å and 142 Å were recorded for I.30E_10MV (Figure 2A) and I.30E_10 (Figure 2B) nanocomposites, respectively. Even though the *d*-spacing distance of the clay was reduced with reduced exposure to vibration, a broader peak was registered for this nanocomposite, representing the presence of smaller clay agglomerates and more disordered orientation [28].

**Figure 3:**
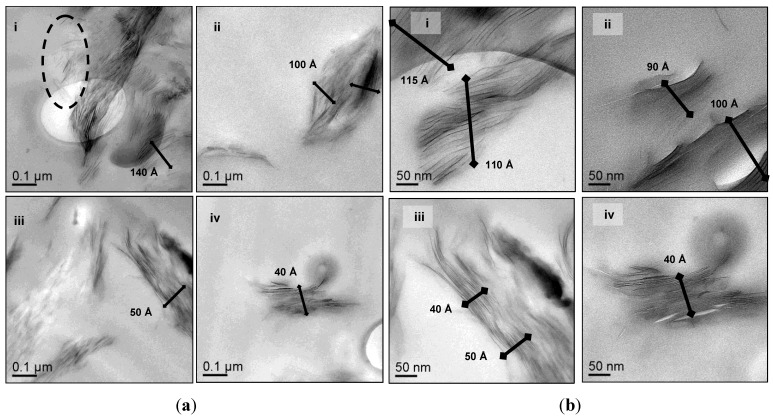
TEM photographs of epoxy/clay nanocomposites (i) I.30E_10; (ii) I.30E_10MV; (iii) C30B_10; and (iv) C30B_10MV at (1.5 × 10^5^)× magnification (**a**); and at (2.5 × 10^5^)× magnification (**b**).

Figure 3 presents representative TEM micrographs at low magnification (A) and high magnification (B) of the I.30E clay structures in the nanocomposites cured at 10 °C/min (i) and at 10 °C/min with vibration (ii). The TEM images suggest that the clay surfactant and rapid heating rate, combined, facilitated the dispersion of individual clay platelets (as observed in the outlined ellipse in A, (i)), although vibration appeared to fracture large agglomerates, so that they were consistently smaller in the resin ((Figure 3A,B, (ii)). The clay morphologies of the C30B clay cured at 10 °C/min (iii) and with vibration (iv) nanocomposites are also shown in Figure 3A,B at the same magnifications. Similar to the I.30E clay, the combination of these processing parameters on the nanocomposites appeared to reduce the size of clay agglomerates, therefore creating a greater distribution of platelets in the resin. It is also noted that a few lighter spheroidal regions were observed in a number of TEM images, which were assumed to be from voids formed during manufacturing, although, overall void content was low.

The average clay *d*-spacing was also measured at a number of locations from the captured TEM images. Particular segment regions where averages were measured in presented micrographs are indicated by the black lines. *D*-spacing values were approximately 110–140 Å for I.30E_10, 90–100 Å for I.30E_10MV, 40–50 Å for C30B_10 and 40 Å for C30B_10MV nanocomposites. The TEM images correlate with the XRD and SAXS results, signifying that the *d*-spacing values of the I.30E clay nanocomposites are greater than for those incorporating C30B clay. Additionally, the decrease in *d*-spacing with vibration was more significant for the I.30E clay.

### 2.2. Optical Microscopy

**Figure 4:**
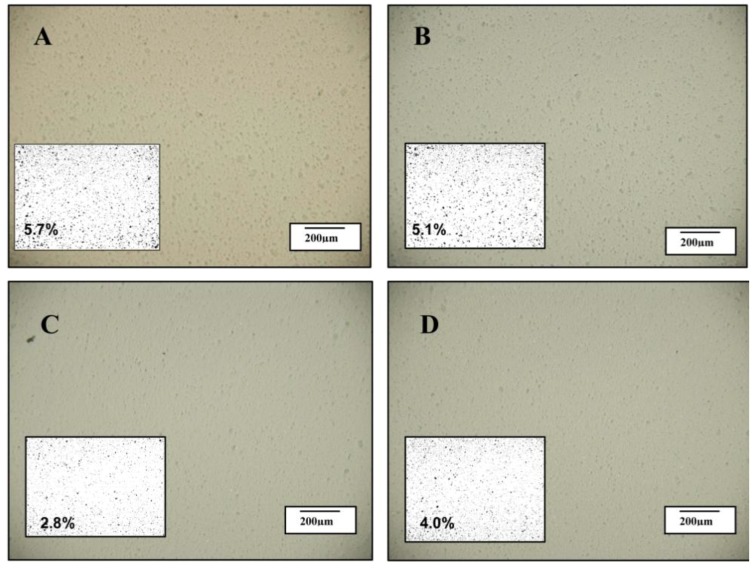
Optical images taken of the following nanocomposites (**A**) I.30E_10; (**B**) I.30E_10MV; (**C**) C30B_10; and (**D**) C30B_10MV.

Optical images of the samples digitised using Image J software were used to quantify the area fraction (%) of the clay agglomerates in the samples. A certain disparity between the separation of platelets and the dispersion of the two clays could be observed on the nano-scale and micron scale. The optical photographs in Figure 4A,B (I.30E nanocomposites) show evidence of clay agglomerates, which consistently appeared larger than for C30B nanocomposites (C and D). When the C30B nanocomposite samples were cured at a rapid heating rate, the area fraction of clay was only 2.8%, although an increase to 4.0% was measured when vibration was applied (see figure insets). The opposite trend was observed in I.30E nanocomposites when processed in the same manner, with the area fraction reducing from 5.7% to 5.1%, suggesting that vibration generated a more homogeneous dispersion on a micron scale.

Therefore, there is some disparity between the optical microscopy results presented in Figure 4 and the XRD, SAXS and TEM results presented in Figure 1, Figure 2 and Figure 3. While the I.30E clays appear to have a dispersed structure on the nano-scale (evident on the XRD and TEM data), platelets are nevertheless incorporated into larger micron-scale agglomerates than the C30B clay nanocomposites (apparent on the optical micrographs). The effect of mechanical vibration was different for each case. The I.30E nanocomposites showed a substantial reduction in micron-scale agglomeration with vibration, while a reduction in *d*-spacing values also suggested a compression of agglomerates on a nano-scale. The C30B nanocomposites, by contrast, displayed a slight improvement in regards to nanoscale agglomeration (XRD and TEM), although they were larger on the micron scale (optical microscopy).

A similar trend was published by Koerner *et al*., between XRD and TEM data, when investigating the dispersion of I.30E and C30B clay in epoxy nanocomposites [29]. It was reported that C30B nanocomposites exhibited a (001) basal reflection >2° of 2*θ*, suggesting an intercalated clay morphology, whereas the diffraction peak for I.30E nanocomposites was detected between 0.5° and 1.0° of 2*θ*, suggesting exfoliated clay structures. TEM images, however, showed that the clay dispersion was more prominent for the C30B nanocomposite. The larger clay *d*-spacing and agglomerates in I.30E nanocomposites were attributed to the catalytic effect of the clay surfactant, which enhanced the crosslink density around the swollen layers. Panagiotis and Triantafyllidis also suggest that the acidic primary onium ions of I.30E catalyse the ring opening of the epoxy and increase the intragallery polymerisation rate [30]. A similar phenomenon is thought to be occurring in the present study and may be due to two main factors, the low viscosity during curing and the differences in clay surfactant chemistries.

### 2.3. Rheology and Surfactant Chemistry

**Figure 5:**
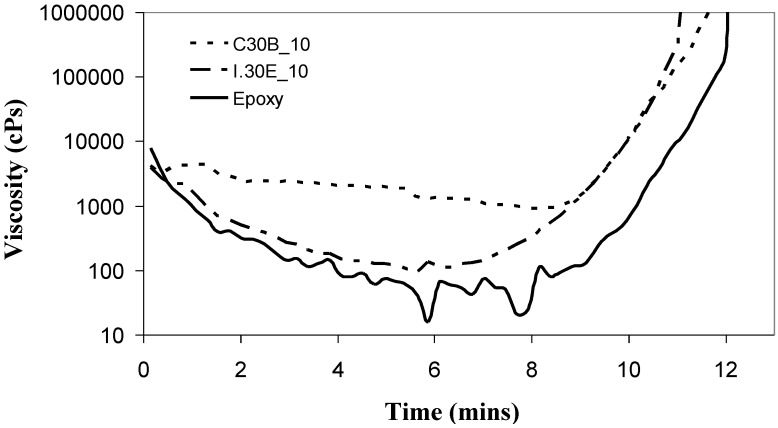
Rheology curves showing that the nanocomposite incorporating C30B clays have a higher viscosity during curing than those with I.30E clays.

The rheological behaviour of the resin mixtures was investigated by simulating the cure cycle (Figure 5). It is noted that the rheometry measurements were conducted directly after the curative was mixed with the epoxy/clay samples at room temperature. At time zero, the rheometer was set to 30 °C, with an identical heating rate to the curing process (10 °C/min). The addition of I.30E clay to the epoxy slightly increased the resin viscosity by ≈20 cPs to ≈90 cPs at its local minimum. It is thought that this minimal increase allows the penetration of the curative, allowing polymerisation between clay galleries to facilitate platelet separation. The relatively large clay *d*-spacing in I.30E nanocomposites might also be influenced by the octadecylamine surfactant in I.30E clay. The acidic nature of this surfactant means it has the potential to catalyse *intra*-gallery polymerisation prior to the epoxy reaction, leading to basal reflections greater than 100 Å [29,30].

**Figure 6:**
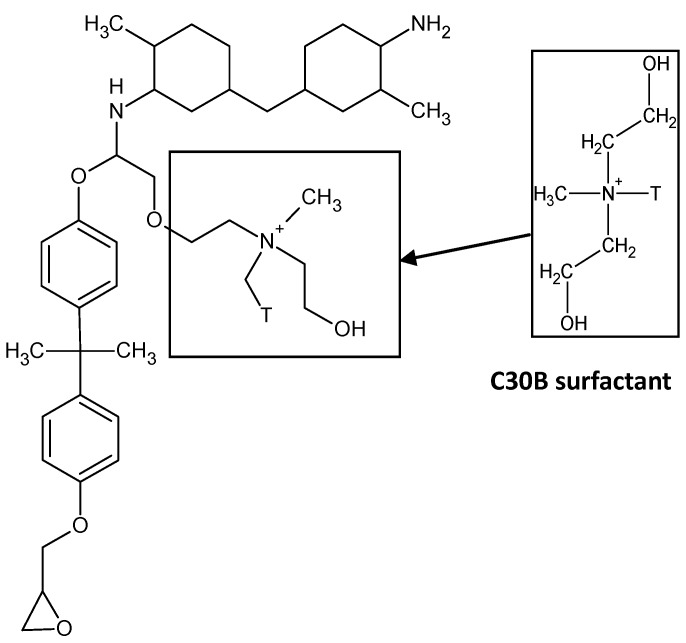
Schematic of the chemical reaction between the hydroxyl group (OH) in the C30B clay and an epoxy end group (CH_2_–O–CH_2_). The epoxy ring opens to react the constituents in order generate polymerisation.

The minimum viscosity of the C30B clay/epoxy prior to gelation is ≈880 cPs, which is 12-times greater than the viscosity of the I.30E clay/epoxy mixture. It is thought that the presence of the quaternary ammonium ions in the C30B clay results in a higher viscosity for the C30B clay/epoxy mixture. This can be attributed to the participation of the functionalised surfaces of C30B clay in the epoxy cure reaction, as schematically shown in Figure 6. To support this, Chen *et al.* [31] reported that quaternary ammonium ions may contribute to an epoxy reaction. Yasmin *et al.* [32] also states that “Cloisite 30B can readily participate in an epoxy reaction”, where technical data from Southern Clay Inc. was referenced. The authors also reported the viscous nature of C30B nanocomposites, although they did not supply rheology data [32]. The octadecylamine groups contained in the surfactant of I.30E clay do not participate in the epoxy curing, but instead, are thought to have a slight catalytic effect [29].

The driving factor in C30B nanocomposites is the epoxy reaction around agglomerates, which only induced intercalated clay morphologies, as detected in the XRD spectrum. The interfacial interaction occurring between the constituents can be summarised from Shi *et al.* [16]. Direct bonding occurs between both clay constituents and epoxy. Specifically, the hydroxylated edge groups contained in the surfactant of the C30B clay can bind to the polymer.

The exposure to mechanical vibration reduced the I.30E clay *d*-spacing from 142 Å to 108 Å, although no significant change was measured in the C30B nanocomposites (Figure 1 and Figure 2). The difference in gallery distances for I.30E and C30B nanocomposites may be due to two intertwining factors: the catalytic surfactant in I.30E clay and low viscosity induced by a rapid heating rate *as well as* mechanical vibration. I.30E nanocomposites were driven by *intra*-gallery polymerisation, where the low viscosity penetrated in between clay galleries, separating the clay platelets. Vibration during curing fractured the clay agglomerates. Since the viscosity could have been reduced further by vibration, due to shear thinning of the polymer, these polymer chains may have flowed out from between the clay platelets, resulting in a compression of the clay galleries. This may have been the cause of the reduced *d*-spacing in I.30E clay galleries. Conversely, the much higher viscosity of the C30B resin mixture possibly limited the gallery expansion. In addition, the bonding of the C30B surfactant to the epoxy reduced the *intra*-gallery expansion, and hence, the *d*-spacing values were smaller. The smaller *d*-spacing in the C30B clay led to smaller micron-scale agglomerates, as evidenced in the optical microscopy images (Figure 4).

It must be noted that the clay gallery distance is not the only factor that may contribute to the different morphologies observed and the potential mechanical properties. As discussed in the previous paper from the authors, clay orientation and agglomerate sizes are also critical parameters [6].

### 2.4. Flexural Tests

**Figure 7:**
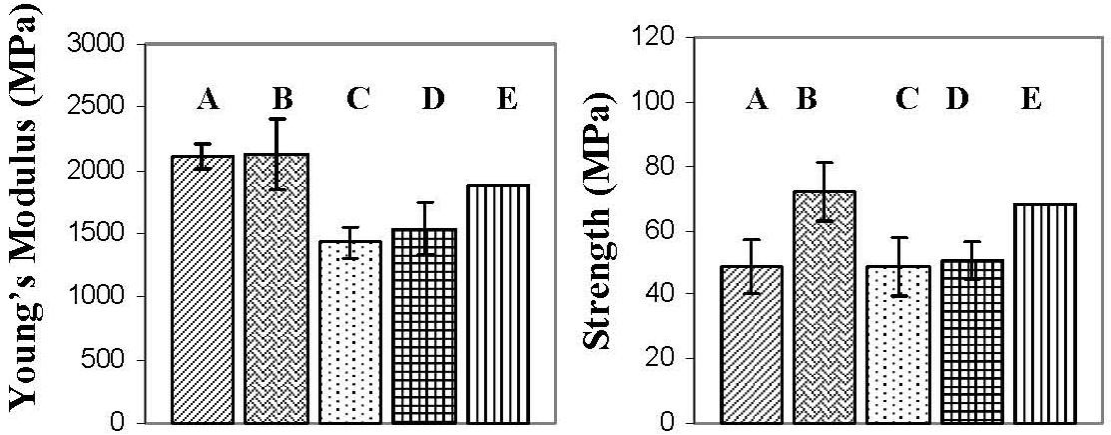
Results from the three point bending tests of the nanocomposites (**A**) I.30E_10; (**B**) I.30E_10MV; (**C**) C30B_10; (**D**) C30B_10MV; and (**E**) Epoxy.

Figure 7 presents the flexural properties of the various nanocomposites. The flexural modulus for the I.30E nanocomposites was increased when compared to epoxy resin, whereas the flexural modulus decreased in the C30B nanocomposites. Others have found that the flexural modulus is enhanced with the incorporation of clay to an epoxy [27,33,34,35,36]. This improvement is often associated with the reinforcement of exfoliated clay, as well as the rule of mixtures theorem, stating that the inclusion of particles should enhance the modulus of the composite [27]. The rule of mixtures does not take in to account the effect of surfactant chemistry and has been used to predict similar results from I.30E and C30B nanocomposites. A study from Ngo *et al.* reported similar differences in modulus data for both I.30E and C30B nanocomposites [27]. Direct clay morphology correlations were not discussed, although scanning electron microscopy (SEM) images illustrated that finer clay dispersion existed with C30B nanocomposites in comparison to I.30E nanocomposites containing the same clay content. Larger clay agglomerates in the nanocomposites were found to increase the flexural modulus, which suggests that a similar phenomenon is occurring between the authors work and that reported by Ngo *et al.* [27]. Ngo *et al.* increased the clay content from 2 wt % to 4 wt %, and the modulus improved, while the clay agglomerate size increased [27]. Therefore, the flexural modulus of epoxy/clay nanocomposites does not increase relative to clay dispersion, but is, instead, correlated with increased agglomeration. These results are also consistent with previous work from the authors, where the flexural modulus decreased as clay disorder became prominent, and agglomerate size was reduced [6].

The flexural strength data is also presented in Figure 7. Unlike the flexural modulus, which is influenced mainly by clay size, loading and morphology [37,38], many factors affect the flexural strength of nanocomposites. These include void content, clay dispersion, interfacial adhesion between the clay and the epoxy and the structure of the materials [27,38]. Given that voids have been reported to act as defects reducing the tensile strength of the nanocomposites [32], optical microscopy was conducted on the epoxy/clay nanocomposite samples (not shown). Minimal void content was observed in the nanocomposites, although C30B nanocomposites did contain visible air pockets on the macro-scale. This could be due to the higher viscosity of the nanocomposites (refer to Figure 5) entrapping air during the mixing and curing process [27,32]. It must be noted that the overall void content in the bulk nanocomposite samples was low. The flexural strength appears to be also critically influenced by clay dispersion in I.30E and C30B nanocomposites. The main distinction arises from the I.30E nanocomposite exposed to mechanical vibration during curing. An increase of 30% was exhibited over all of the other nanocomposites, although the size of the micron clay agglomerates was smaller in C30B nanocomposites than the I.30E nanocomposites. Taking into account the void content in the C30B nanocomposites, porosity and agglomerate size factor together as a combined effect on the strength. Although the strength would be expected to increase, due to the reduction in large agglomerates, the impact of porosity negated this. Therefore, it is thought that the smaller agglomerates would increase in strength if the C30B nanocomposite was not prone to porosity.

Even though strength properties are dependent on many factors, morphology results show that smaller clay agglomerates enhance nanocomposite strength (when taking porosity into consideration). It is thought that agglomerates need to be reduced beyond some critical maximum size to enhance strength. As shown in the optical microscopy data (Figure 4), clay agglomerates observed on a micro-scale should be no higher than an area fraction of approximately 5.1% for this to occur. Therefore, processing variables and clay surfactants may contribute to flexural properties.

### 2.5. Dynamic Mechanical Analysis

**Figure 8:**
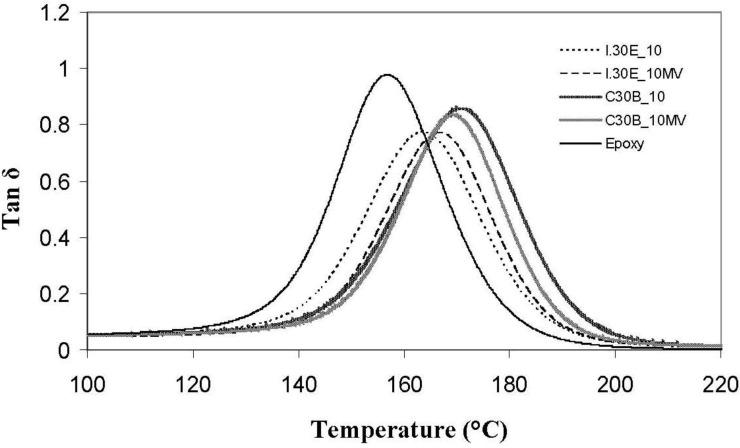
T_g_ measured from tanδ peaks for I.30E and C30B nanocomposites.

Dynamic mechanical analysis (DMA) traces of various clay surfactants in C30B and I.30E nanocomposites, as well as processing variables, are displayed in Figure 8. In comparison to the epoxy resin (158 °C), the T_g_ values (tanδ peak) of the nanocomposites were detected at elevated temperatures of up to 172 °C, implying that the polymer molecular motion is restricted, due to the addition of nanoclay filler. The T_g_ measured for I.30E nanocomposites was ≈165 °C, which is ≈10 °C higher than that of the resin. C30B clay restricted molecular motion further, as the functional groups on the clay platelets participate in the cure reaction, and the polymer chains are, therefore, tethered to platelets. Molecular motion can be hindered, due to a number of reasons, including greater interfacial adhesion between the epoxy and clay, but also the clay morphology in the nanocomposites. All of these factors may have contributed to the observed increase in glass transition temperature as measured from the tanδ peak. Therefore, this phenomenon was believed to range from contributing aspects, such as a lack of surrounding entanglements, a change in chemistry and un-reacted resin plasticisation (interphase between epoxy/clay surfactant).

Even though dispersion is not the only factor to influence variations in T_g_, C30B nanocomposites consist of smaller agglomerates, which are thought to enhance the T_g_ significantly in comparison to epoxy (≈170 °C to 158 °C, respectively). Given that the T_g_ of the nanocomposites increased, a plasticised epoxy/clay surfactant interphase appears to be minimal, as plasticisation generally results in a negative shift of tanδ. Disparity surrounding the glass transition temperature indicates that the variation in clay dispersion and epoxy/clay interfaces have an impact on the network relaxation [29]. The fact that the C30B nanocomposite T_g_ was higher than that of the I.30E nanocomposite signifies that greater interfacial adhesion exists between C30B clay and epoxy, where molecular mobility is restrained. A similar finding was reported by Panagiotis and Triantafyllidis, whereby a reduction in T_g_ for their I.30E nanocomposites was attributed to the octadecylammonium chains causing a plasticising effect at the epoxy clay interface [30]. Becker *et al*. reported that an “adsorbed layer” effect usually enhances the T_g_ by means of polymer chains attached to the surfactant in the clay [34]. The notion that the C30B clay surfactant can potentially react with the epoxy to enhance the T_g_, supports the analysis of assumed chemical reaction mechanism (as shown schematically in Figures 6).

Variations in processing techniques did not seem to significantly affect the nanocomposite T_g_’s, although surfactant chemistry in the clay was shown to have an effect. I.30E nanocomposites obtained a reduction in the height of the tanδ peak, where I.30E clay stiffens resin more so than C30B clay. When referring to the flexural modulus of these nanocomposites (Figure 7), I.30E clay offered greater reinforcement to the resin in comparison to C30B clay. Therefore, an increase in the flexural modulus for I.30E nanocomposites can be correlated with a more significant reduction in the tanδ peak. The smaller clay agglomerates processed with C30B clay did not reinforce the resin, but rather, the smaller agglomerates reduced the flexural modulus. Conversely, I.30E clay may enhance stiffness in resin, since the clay agglomerates were larger.

## 3. Experimental Section 

### 3.1. Materials

Diglycidyl ether of bisphenol A/epichlorohydrin (DGEBA)-based epoxy resin (Epon 828) was obtained from Chemiplas Australia Pty Ltd. A cycloaliphatic polyamine derived from 2,2′-dimethyl-4,4′-methylenebis (Aradur 2954) was purchased from Meury Enterprises Pty Ltd. Cloisite 30B (C30B) clay was acquired from Southern Clay Products Inc. and is functionalised with an alkyl quaternary ammonium salt. Nanomer I.30E clay is modified with an octadecylammonium salt and purchased from Nanocor^®^. Table 1 summarises the chemical structures of the organic surfactants for I.30E and C30B clays. Both clays are relatively inexpensive and obtain good intercalation chemistry with epoxy resins. The epoxy resin was degassed overnight to eliminate any moisture uptake in the hydrophilic materials, which would reduce the void content present in the cured epoxy/clay nanocomposites.

### 3.2. Preparation of Epoxy/Clay Nanocomposites 

Organoclay corresponding to 5 wt % of the epoxy resin (Epon 828 + Aradur 2954) was combined with a given amount of Epon 828 resin and heated to 70 °C. The mixture was stirred for 2 h using a magnetic stirrer plate fitted with a temperature probe to control temperature fluctuations during the period that the clay would swell in the resin. A Hielscher UIP1000-230 ultrasonic processor operating at a frequency of 20 kHz was used to generate ultrasonic waves with an amplitude of 80 µm peak-to-peak through the epoxy/clay mixture for 30 min. This sonication technique was utilised to facilitate the dispersion of clay. The mixture was situated in an ice bath, and an ultrasonic pulsing cycle of 2 s on and 4 s off was used. The temperature of the mixtures was then reduced to 50 °C, where a stoichiometric quantity of the curative was added and degassed for 20 min prior to casting in a mould.

### 3.3. Curing Protocol

Quickstep™ QS5 (from Quickstep Technologies Pty Ltd.) was employed to manufacture epoxy resin and epoxy/clay nanocomposites using a rapid heating rate of 10 °C/min combined with mechanical vibration. This heating rate is significantly higher than that of conventional autoclave and oven curing technologies, which are ~3 °C/min [6]. Samples were heated to 130 °C at 10 °C ± 2 °C/min and held isothermally for 30 min. The laminates were exposed to mechanical vibration at a frequency of 4 Hz for 10 min during the heat up stage of the cure profile. The pressure of the vacuum was maintained at −70 kPa during cure. These conditions prevented the mixtures from boiling. Samples are referenced according to the clay used and whether vibration was employed in the cure cycle. For example, C30B_10 refers to the nanocomposite containing Cloisite 30B clay and cured at a heating rate of 10 °C/min. I.30E_10MV indicates that vibration was utilised during the heat up of the nanocomposites containing I.30E clay.

### 3.4. Rheometry

Viscosity profiles were measured using an ARES (TA Instrument) rheometer. Approximately 2 g of a liquid sample were placed between the two parallel plates, 25 mm in diameter with a gap of 1 mm. For these tests, the curative was stirred into the epoxy/clay mixtures at room temperature, and these were not degassed (as conducted in the preparation for the Quickstep™ curing protocol). Tests were conducted in dynamic temperature ramp mode, where the temperature ranged from 30 °C to 130 °C, with a heating rate of 10 °C/min. The strain and frequency used were 10% and 1 rad/s, respectively.

### 3.5. X-ray Diffraction

Diffraction patterns were obtained using a PANalytical X’Pert Pro Diffractometer with Cu Kα radiation (λ = 1.54184 Å) operating at 45 kV and 40 mA with a divergent slit size of 0.03°. Scans were acquired in continuous mode over a range of 1°–10° (2*θ*) with a step size of 0.033, with the sample spinning. Prior to analysis, the cured samples were ground flat using P1200 Silicon Carbide (SiC) wet/dry paper and cleaned with ethanol.

### 3.6. Small Angle X-ray Scattering 

Small angle X-ray scattering measurements were performed on a Bruker Nanostar SAXS camera, with pin-hole collimation for point focus geometry. All tests were conducted using a copper rotating anode (0.3 mm filament) operating at 45 kV and 110 mA. This was fitted with cross-coupled Gobel mirrors, resulting in a Cu Kα radiation wavelength of 1.54184 Å. The SAXS camera was fitted with a Hi-star 2D detector. The sample to detector distance was chosen to be 1070 mm, which provided a q-range of 0.008 to 0.22 Å^−1^. The optics and the sample chamber were under vacuum to minimise air scatter. Cured samples were polished flat to within 1 mm thickness prior to testing. An epoxy resin sample was scanned to subtract any background scattering noise and used as a baseline for analysis.

### 3.7. Optical Microscopy

Samples were cast in 30 mm diameter metallographic mounts and analysed on an Olympus BX51M fitted with a DP70 High resolution camera. The cross-sectional images were examined using Image J analysis software to calculate the area fraction (%) of the clay entities in the nanocomposites. Optical images were converted to binary images (black and white) during thresholding. Clay constituents are represented by the black pixels.

### 3.8. Transmission Electron Microscopy 

Epoxy/clay nanocomposites were examined using a JEOL 2010F field emission TEM, fitted with a Gatan Imaging Filter (GIF) and operating at an accelerating voltage of 200 keV. Samples were prepared at room temperature using a Leica Ultracut UCT ultramicrotome, where they were trimmed and sectioned with Diatome diamond knives. Sections were cut to a thicknesses of 80–100 nm and collected on a copper grid with a carbon support.

### 3.9. Flexural Tests

Solid samples were pre-conditioned in a controlled temperature (20 ± 2 °C) and relative humidity (65% ± 2%) environment prior to testing. Three point bending tests were conducted under Method I of ASTM D790. A minimum of 5 samples per batch were fractured using a Lloyd LR30K tensile instrument fitted with a 1 kN load cell at a crosshead speed of 1.28 mm/min. The geometries of the samples were 70 mm × 10 mm × 3 mm, according to the ASTM standard D790.

### 3.10. Dynamic Mechanical Analysis 

Samples were tested on a DMA Q800. To remain consistent, the samples used for flexural tests were then reduced to a thickness of 2 mm to comply with a single cantilever beam method for ASTM E1640-99. The glass transition temperature (T_g_) was measured using a temperature scan between 30 °C and 250 °C, an amplitude strain of 0.5%, a frequency of 1 Hz and a heating rate 2 °C/min.

## 4. Conclusions 

This study highlights three main outcomes; (i) the combination of high amplitude sonication, a rapid heating rate and mechanical vibration during cure enhance the dispersion of I.30E clay in epoxy resin; (ii) the importance of using a number of complementary techniques to completely characterise localised and spatial clay morphology in a nanocomposite on a nano- and micron scale; and (iii) structure-property relationships can be observed when not obscured by the effect of large agglomerates.

Surfactant chemistry plays an important role in the dispersion of platelets. The gallery spacings in the C30B nanocomposites were influenced by high viscosity and surfactant-epoxy interface bonding, leading to extra gallery polymerisation and *d*-spacing values <40 Å. Conversely, lower viscosity and a catalytically-driven surfactant on the I.30E clay led to I.30E nanocomposites exhibiting greater nanoscale dispersion (larger *d*-spacing). Importantly, mechanical vibration reduced micron-sized agglomerates in the I.30E nanocomposites and enabled the enhancement in flexural strength and thermo-mechanical properties. The synthesis and characterisation of epoxy/clay nanocomposites is necessary to understand structure-property relationships. Techniques, including XRD, SAXS, TEM and optical microscopy, are essential to characterise these morphologies over various scales; otherwise, the nature of the dispersion may easily be misinterpreted.
